# Coronavirus disease 2019 death prediction by electrocardiographic abnormalities and elevated D-dimer levels

**DOI:** 10.3389/fcvm.2022.948347

**Published:** 2022-09-28

**Authors:** Jing Chen, Yina Wang, Jingyi Wang, Lie Chen, Qiushi Luo, Bei Wang, Xingwei He, Xuefei Li, Huakun Zuo, Ping Zuo, Xiaoyun Yang

**Affiliations:** ^1^Division of Cardiology, Department of Internal Medicine, Tongji Hospital, Tongji Medical College, Huazhong University of Science and Technology, Wuhan, China; ^2^Department of Rheumatology and Immunology, Tongji Hospital, Tongji Medical College, Huazhong University of Science and Technology, Wuhan, China; ^3^Wuhan National High Magnetic Field Center, Huazhong University of Science & Technology, Wuhan, China

**Keywords:** COVID-19, electrocardiogram, d-dimer, in hospital, death prediction

## Abstract

**Background:**

Electrocardiography (ECG) plays a very important role in various cardiovascular diseases and elevated D-dimer in serum associated with thrombosis. In patients with coronavirus disease 2019 (COVID-19), immense pieces of evidence showed that ECG abnormalities or elevated D-dimer in serum occurred frequently. However, it remains unclear whether ECG abnormalities combined with elevated D-dimer could be a new risk predictor in patients with COVID-19.

**Methods and results:**

This retrospective cohort study enrolled 416 patients with COVID-19 at Wuhan Tongji Hospital from 1 February to 20 March 2020. ECG manifestations, D-dimer levels, and in-hospital deaths were recorded for all patients. Logistic regression analysis was performed to examine the association between ECG manifestations and in-hospital mortality in patients with elevated D-dimer levels. In patients hospitalized for COVID-19, ST-T abnormalities (34.3%) were the most frequent ECG manifestations, whereas sinus tachycardia (ST) (13.3%) and atrial arrhythmias with rapid rhythms (8.5%) were the two most common cardiac arrhythmias. Compared to severely ill patients with COVID-19, ST-T abnormalities, ST and atrial arrhythmias (*p*<0.001) with rapid rhythms, D-dimer levels, and in-hospital deaths were significantly more frequent in critically ill patients with COVID-19. Moreover, elevated D-dimer levels were observed in all the patients who died. In the subgroup of 303 patients with elevated serum D-dimer levels, the patient's age, the incidence of ST-T abnormalities, ST, atrial fibrillation (AF), and atrial premature beat were significantly higher than those in the non-elevated D-dimer subgroup. Multivariate logistic regression analysis further revealed that ST and AF were risk factors for in-hospital mortality in COVID-19 patients with elevated D-dimer levels.

**Conclusions:**

ECG abnormalities and elevated D-dimer levels were associated with a higher risk of critical illness and death in patients hospitalized for COVID-19. ECG abnormalities, including ST and AF, combined with elevated D-dimer levels, can be used to predict death in COVID-19.

## Introduction

The novel coronavirus disease 2019 (COVID-19) infection has potential effects on the cardiovascular system, with excessive inflammation and immune responses that could induce coronary vasculitis (1), cardiac injury, cardiac arrhythmias (2), and heart failure (3). Electrocardiography (ECG) is an important examination for the diagnosis of heart rhythm disturbances and other myocardial diseases in patients with COVID-19 (4). The electrocardiographic features of patients with COVID-19 pneumonia have been defined in several studies (5, 6). ECG abnormalities in patients with COVID-19 mainly include ST-T abnormalities caused by acute coronary syndromes, myocarditis, acute pericarditis, electrolyte disturbances, rhythm disorders, and pulmonary embolism (PE)-related manifestations (6, 7). A previous study demonstrated that right ventricular strain patterns on ECG are tightly associated with increased mortality in PE (8). On ECG, right ventricular strain pattern (9), fragmented QRS (10), prolonged QT (11), abnormal axis (12), and left bundle branch block (12) have all been associated with poor outcomes and a higher risk of mortality in COVID-19. As ECG is an easy and accessible method, ECG monitoring of patients with COVID-19 can be used as an essential screening tool for identifying high-risk patients.

D-dimer is a product of fibrin degradation and is a marker of fibrinolysis activation. The combination pre-test probability of venous thromboembolism with D-dimer levels <1000 μg/L can be used to evaluate the risk of venous thromboembolism and PE (13). Some studies demonstrated that elevated D-dimer was closely associated with mortality in patients with COVID-19 (14, 15). Furthermore, COVID-19 patients with elevated D-dimer levels exhibited a high prevalence of PE (16). ECG abnormalities in acute PE have been characterized by mounting evidence and play a valuable role in the diagnosis of PE (17). However, ECG abnormalities combined with elevated D-dimer in mortality prediction of COVID-19 remain unexplored.

Hence, we assessed the ECG abnormalities as well as elevated D-dimer levels in COVID-19. Specifically, we investigated whether the abnormalities in ECG together with elevated D-dimer levels were potential risk predictors for mortality in COVID-19.

## Materials and methods

This was a retrospective, observational study performed on patients diagnosed with COVID-19 admitted to Optical Valley Campus and the Sino-French New Campus of Tongji Hospital of Huazhong University of Science and Technology in Wuhan from 1 February 2020 to 20 March 2020. All consecutive patients who underwent ECG examination during this time frame were included. Optical Valley Campus and the Sino-French New Campus of Tongji Hospital were urgently reconstructed and specially designated hospitals to treat severely or critically ill patients with COVID-19. According to the Guidance for Corona Virus Disease 2019 (7th edition), patients with COVID-19 were diagnosed and divided into the critically ill and severely ill groups (18). This research was approved by the Institutional Review Board for Human Studies, Tongji Hospital, Huazhong University of Science and Technology (TJ-20200140).

Data from patients, including age, sex, medical history, ECG and laboratory examinations, clinical classification, and outcomes during hospital admission, were collected and analyzed. Clinical data were monitored until 20 March 2020.

The 12-lead routine ECG was recorded at an amplification of 10 mm/mV and a paper speed of 25 mm/s. All ECG examinations were recorded by one investigator who was double-blinded to all other clinical processes. The following abnormal signs in ECG were especially focused on: (1) sinus tachycardia (ST) (heart rate >100 bpm). (2) ST-T abnormalities: the elevation of ST segment was defined as ST 0.06 s after QRS complex in V_2_ and V_3_ leads and ≥0.1 mV in ≥2 other contiguous leads. The ST segment was elevated by ≥0.2 mV in men over 40 years and by ≥0.25 mV in men below 40 years. In women, the elevation of the ST segment was ≥0.15 mV (19). The evaluation of depression on the ST segment was defined as horizontal depression >0.05 mV or downsloping depression >0.05 mV from the isoelectric line, 0.06 s after QRS complex ending, and negative T waves were considered negative depth >0.10 mV in 2 contiguous leads (19). (3) incomplete (IRBBB) or complete right bundle branch block (CRBBB). (4) atrial flutter (AFL). (5) atrial fibrillation (AF). (6) abnormal Q wave. (7) left ventricular high voltage; (8) degree I atrioventricular block; (9) atrial premature beat; (10) ventricular premature beat; (11) atrial tachycardia; and (12) sinus bradycardia. ECG abnormalities in the Daniel score system include ST (heart rate >100 bpm), IRBBB or CRBBB, T-wave inversion, and the S_1_Q_3_T_3_ complex (8). The ST (heart rate >100 bpm) and IRBBB scores were recorded at two points. The CRBBB score was recorded as three points. According to the magnitude of the T-wave inversion in leads V_1_, V_2_, and V_3_, the score was recorded as 0–3 points. The score of S_1_Q_3_T_3_ components indicated that the S wave in lead I was 0 points, and the Q wave and T-wave inversion in lead III were both one point.

Serum D-dimer levels were examined in a Tongji Hospital laboratory at the time of admission. D-dimer levels >500 μg/L were considered abnormal.

### Statistical analysis

The ECG distributions in patients with COVID-19 were further analyzed using a descriptive computing program with an SPSS multiple response set. Continuous variables with normal distributions are shown as mean ± SD. Additionally, comparisons between the two groups were analyzed using Student's *t*-test. Moreover, continuous variables with skewed distributions are represented by the median (quartile range), and the significance between groups was examined using the Mann-Whitney test. Categorical data are expressed as numbers or percentages and were compared using the χ^2^ test. A two-sided significance level of 0.05 was applied in all hypothesis tests. Logistic regression analyses were used to determine baseline characteristics and ECG signs of mortality in the subgroups of elevated and non-elevated D-dimer levels. For univariate and multivariate analyses, variables with a 2-sided *p*-value <0.05 were included. All statistical analyses were performed using SPSS (version 18, Chicago, NY, USA).

## Results

**Figure 1:**
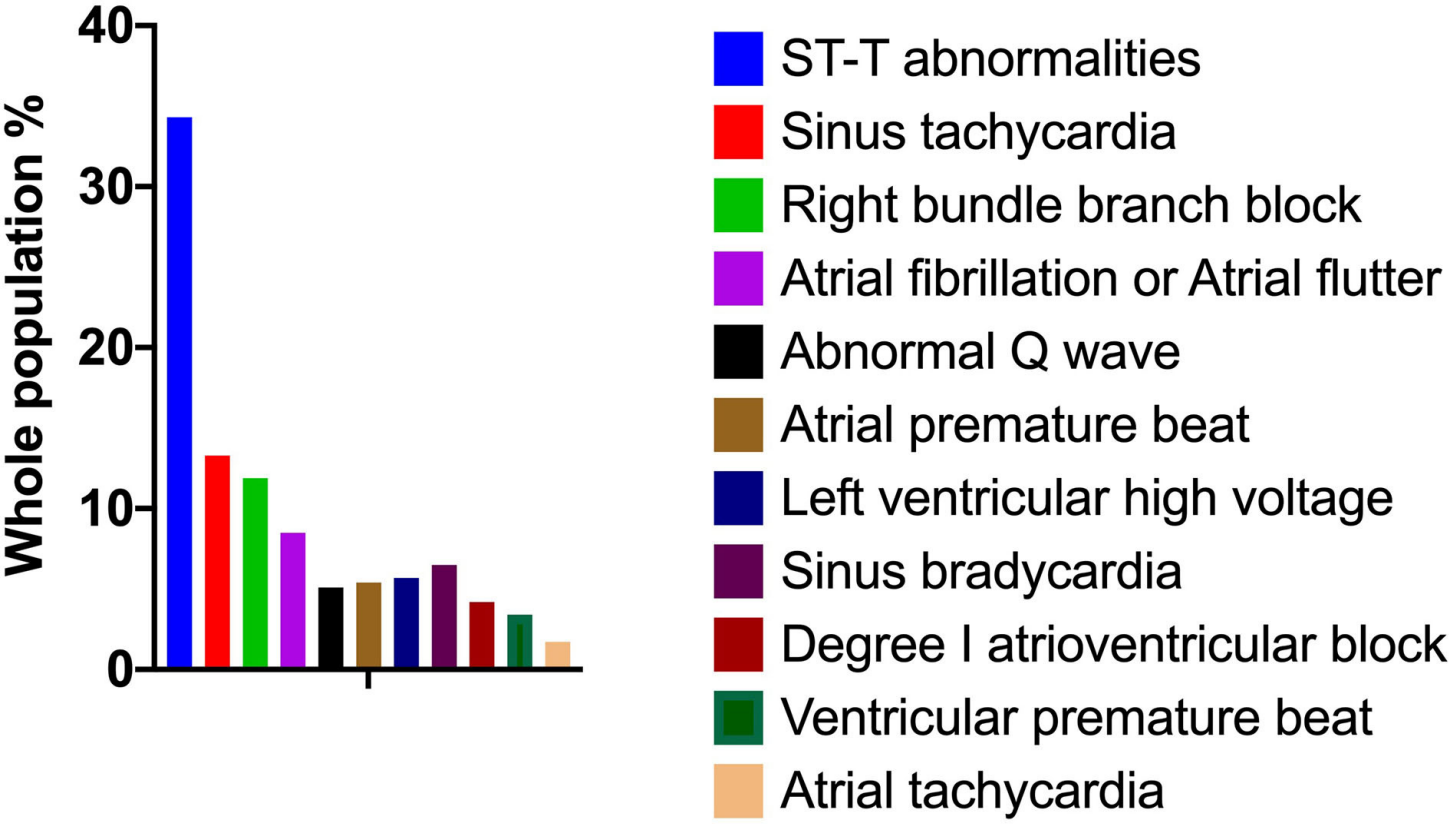
Electrocardiogram characteristics of 416 patients hospitalized for COVID-19. The frequencies of ECG characteristics in patients with COVID-19 were evaluated by a descriptive computing program using an SPSS multiple response set. COVID-19, coronavirus disease 2019; ECG, electrocardiography.

In this study, we analyzed 416 consecutive patients hospitalized for COVID-19. The frequencies of the ECG characteristics were evaluated. According to the descriptive computing program using an SPSS-multiple response set (Figure 1), ST-T abnormalities (34.3%) were the most frequent abnormality in these hospitalized patients. ST (heart rate >100 bpm) (13.3%) was the most prevalent cardiac arrhythmia encountered. In addition, the RBBB, AF, and AFL increased by 11.9 and 8.5%, respectively. Of all the ECG characteristics, the least occurring was ventricular premature beats (1.7%).

The 416 patients with COVID-19 were divided into critically and severely ill groups. As seen in Table 1, the average age of critically ill patients (67.4 ± 14.4 years) was older than that of severely ill patients (62.1 ± 14.0 years). The proportion of men among severely ill patients (178, 59.1%) was higher than that among critically ill patients (35, 30.4%). Hypertension, coronary heart disease, arrhythmia, and stroke were much more frequent among critically ill patients [60 (52.2%), 21 (18.3%), 6 (5.2%), and 9 (7.8%), respectively] than among severely ill patients [108 (35.9%), 29 (9.6%), 3 (0.9%), and 6 (2.0%), respectively] (Table 1). D-dimer levels in critically ill patients (111, 96.5%) were significantly higher than those in the severely ill group (192, 63.8%) (Table 1).

**Table 1:** Comparison of the characteristics of critically ill patients with COVID-19 and severely ill patients with COVID-19.

	**Critically ill patients (*n* = 115)**	**Severely ill patients (*n* = 301)**	***p*-value**	**Chi-square**
Age (years)	67.4 ± 14.4	62.1 ± 14.0	<0.001	
Male, *n (%)*	35 (30.4)	178 (59.1)	<0.001	27.43
**Medical history**				
Diabetes mellitus, *n (%)*	33 (28.7)	59 (19.6)	0.046	3.995
Hypertension, *n (%)*	60 (52.2)	108 (35.9)	0.003	9.17
Cornary heart disease, *n (%)*	21 (18.3)	29 (9.6)	0.016	5.855
Arrhythmia, *n (%)*	6 (5.2)	3 (0.9)	0.008	7.003
Stroke, *n (%)*	9 (7.8)	6 (2.0)	0.004	8.145
D-dimer≥500ug/L, *n (%)*	111 (96.5)	192 (63.8)	<0.001	121.5
**ECG characteristics**				
ST-T abnormalities, *n (%)*	57 (49.6)	64 (21.3)	<0.001	32.32
Sinus tachycardia, *n (%)*	30 (26.1)	17 (5.6)	<0.001	34.69
Right bundle branch block, *n (%)*	15 (13)	27 (9.0)	0.124	2.372
Atrial arrhythmias (rapid rhythms), *n (%)*	25 (21.7)	11 (3.7)	<0.001	34.43
Atrial fibrillation	16 (13.9)	9 (3.0)	<0.001	15.39
Atrial flutter	4 (3.5)	1 (0.3)	0.008	6.935
Atrial tachycardia	5 (4.3)	1 (0.3)	0.002	9.439
Abnormal Q wave, *n (%)*	11 (9.6)	7 (2.3)	0.001	10.54
Atrial premature beat, *n (%)*	10 (8.7%)	9 (3.0)	0.013	6.215
Left ventricular high voltage, *n (%)*	8 (7.0)	12 (4.0)	0.177	1.821
Sinus bradycardia, *n (%)*	7 (6.1)	16 (5.3)	0.758	0.095
Degree I atrioventricular block, *n (%)*	5 (4.3)	10 (3.3)	0.616	0.252
Ventricular premature beat, *n (%)*	6 (5.2)	6 (2.0)	0.079	3.087
In-hospital mortality, *n (%)*	46 (40.0)	1 (0.3)	<0.001	130.6

ST-T abnormalities (57, 49.6%) were the most prevalent ECG abnormalities, and ST (heart rate >100 bpm) (30, 26.1%) was the most frequent cardiac arrhythmia in the critically ill group. The proportions of these ECG characteristics in critically ill patients were significantly more prevalent when compared to those in the severely ill group [64 (21.3%) and 17 (5.6%), respectively] (Table 1). The second most prevalent cardiac arrhythmia in critically ill patients was atrial arrhythmia, which was characterized by rapid rhythms, including AF, AFL, and atrial tachycardia. Atrial arrhythmias with rapid rhythms were markedly more prevalent in critically ill patients (25, 21.7 %) than in severely ill patients (11, 3.7%). Other prevalent ECG characteristics in critically ill patients include RBBB, abnormal Q wave, atrial premature beat, left ventricular high voltage, sinus bradycardia, degree I atrioventricular block, and ventricular premature beat. In addition, abnormal Q waves and atrial premature beats were significantly more frequent in critically ill patients [11 (9.6%) and 10 (8.7%), respectively] than in severely ill patients [7 (2.3%) and 9 (3.0%), respectively]. In-hospital mortality was significantly higher in critically ill patients (46, 40.0%) than in severely ill patients (1, 0.3%) (Table 1).

D-dimer levels were evaluated between the surviving and dead groups. Notably, 47 (100%) patients who died of COVID-19 had elevated D-dimer levels (*p* < 0.001) compared with the surviving group (Table 2).

**Table 2:** Comparison of in-hospital death in patients with elevated D-dimers and non-elevated D-dimers.

	**Dead (*n* = 47)**	**Survival (*n* = 369)**	**Chi-square**	***p*-value**
Elevated D-dimers, *n* (%)	47 (100)	256 (69.4%)	19.76	<0.001
Non-elevated D-dimers, *n* (%)	0 (0)	113 (30.6%)		

**Figure 2:**
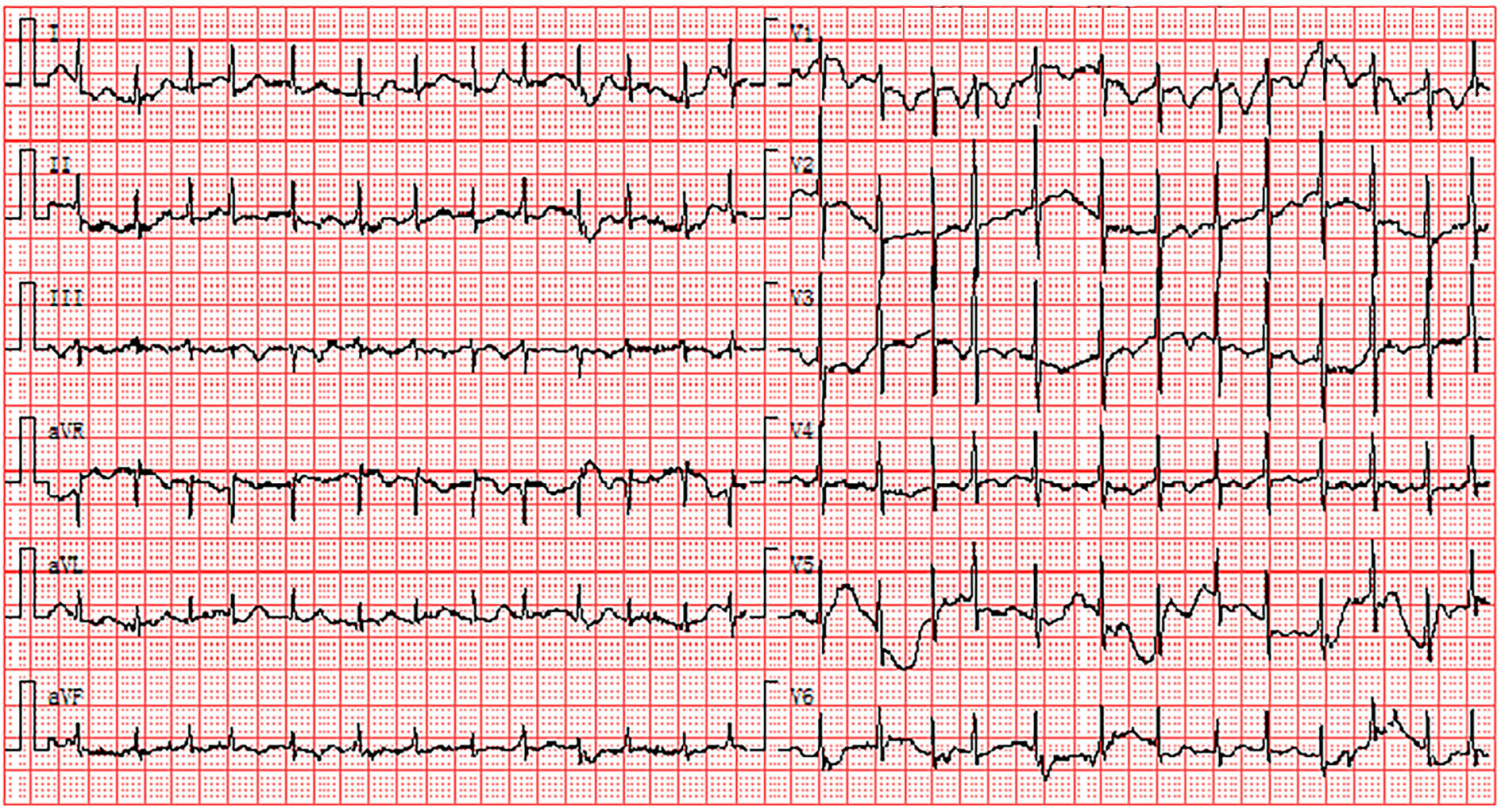
Electrocardiogram abnormalities associated with elevated D-dimers: the existence of AF and ST-T abnormalities. AF, atrial fibrillation.

To further investigate the clinical characteristics of these patients with elevated D-dimers, we compared the age, proportion of men, and ECG characteristics of patients with and without elevated D-dimer levels (Table 3). Compared with patients without elevated D-dimers, patients with elevated D-dimers were significantly older (66.8 ± 12.9 vs. 54.9 ± 14.2). In addition, compared with patients with normal D-dimer levels, ECG findings showed that ST-T abnormalities [100 (33%) vs. 21 (18.6%)], ST (sinus rate >100 bpm) [43 (14.2%) vs. 4 (3.5%)], AF [23 (7.6%) vs. 2 (1.8%)], and atrial premature beat [18 (5.9%) vs. 1 (0.8%)] were significantly increased in patients with elevated D-dimer levels (Figure 2, Table 3).

**Table 3:** Comparison of the characteristics of patients with elevated D-dimers and patients with non-elevated D-dimers.

	**Elevated D-dimers (*n* = 303)**	**Non-elevated D-dimers (*n* = 113)**	***p*-value**	**Chi-square**
Age (years)	66.8 ± 12.9	54.9 ± 14.2	<0.001	
Male	156 (51.5)	47 (41.6)	0.073	3.223
**ECG characteristics**				
ST-T abnormalities, *n (%)*	100 (33)	21 (18.6)	0.0057	7.629
Sinus tachycardia, *n (%)*	43 (14.2)	4 (3.5)	0.002	9.318
Right bundle branch block, *n (%)*	32 (10.6)	10 (8.8)	0.606	0.266
Atrial arrhythmias (rapid rhythms), *n (%)*	34 (11.2)	2 (1.8)	0.002	9.3
Atrial fibrillation	23 (7.6)	2 (1.8)	0.032	4.564
Atrial flutter	5 (1.7)	0	0.17	1.887
Atrial tachycardia	6 (2.0)	0	0.132	2.27
Abnormal Q wave, *n (%)*	16 (5.3)	2 (1.8)	0.118	2.45
Atrial premature beat, *n (%)*	18 (5.9)	1 (0.8)	0.035	4.44
Left ventricular high voltage, *n (%)*	12 (4.0)	8 (7.1)	0.186	1.75
Sinus bradycardia, *n (%)*	18 (5.9)	5 (4.4)	0.631	0.23
Degree I atrioventricular block, *n (%)*	13 (4.3)	2 (1.8)	0.22	1.504
Ventricular premature beat, *n (%)*	10 (3.3)	2 (1.8)	0.407	0.688

**Figure 3:**
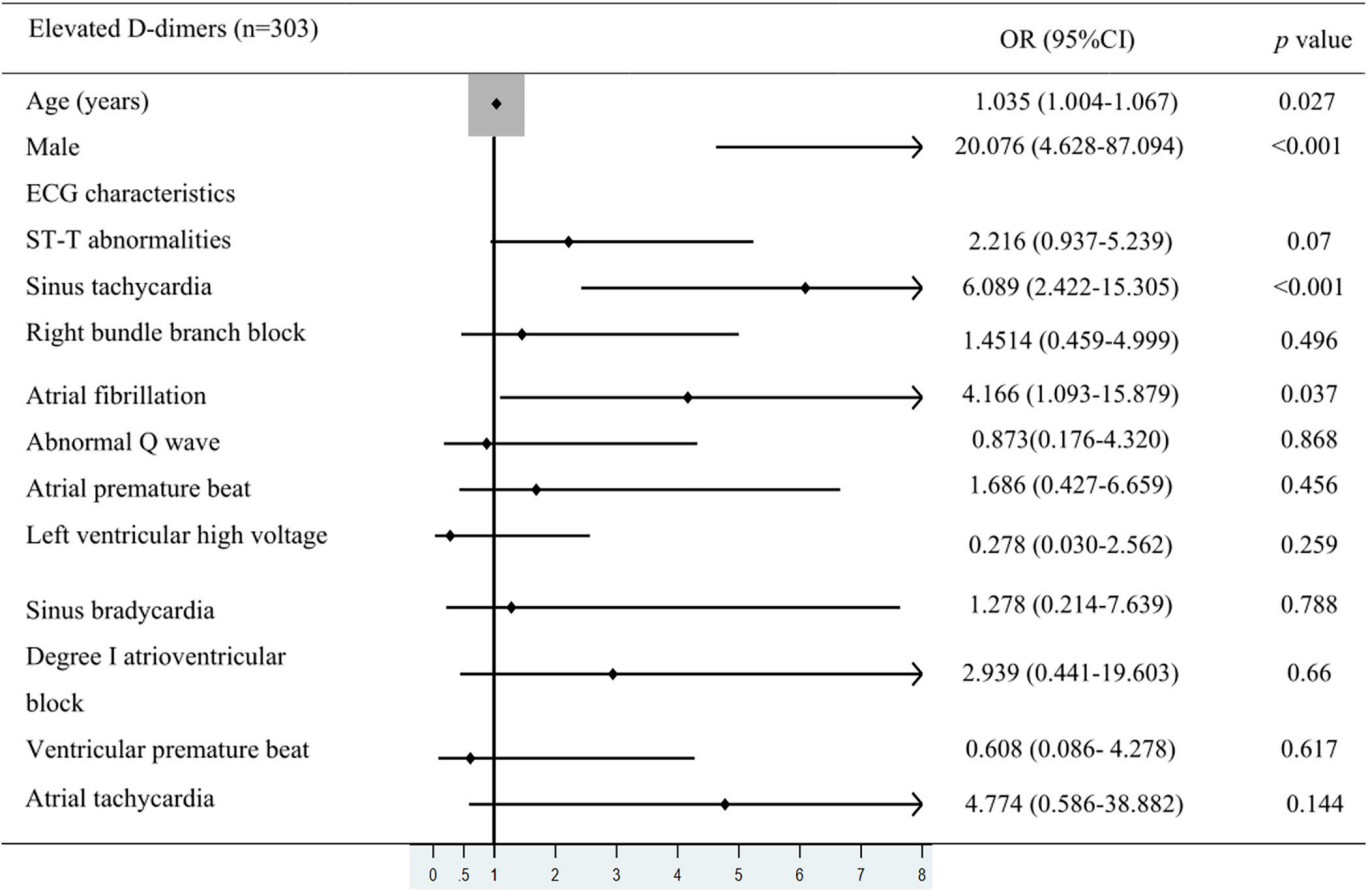
Multivariate logistic regression analysis. The values were included in multivariate logistic regression analysis such as in-hospital death, patients' mean age, the proportion of men, and ECG characteristics in COVID-19 patients with elevated D-dimer levels.

To further clarify the relationship between ECG characteristics and in-hospital death in patients with elevated D-dimer levels, multivariate logistic regression analysis and Forest plots were used. In patients with elevated D-dimer levels, 47 died and 256 survived. Values such as in-hospital death, patients' mean age, the proportion of men, and ECG characteristics were included in the multivariate logistic regression analysis. Odds ratios (ORs) with 95% confidence intervals (CI) are shown in Figure 3. ST (sinus rate >100 bpm) (OR: 6.089, 95% CI: 2.422–15.305, *p* < 0.001) and AF (OR: 4.166, CI: 1.093–15.879, *p* = 0.037) were independent risk factors associated with in-hospital mortality in the elevated D-dimer group (Figure 3). Besides the ECG signs, according to the multivariate analysis, the average age (OR: 1.035, CI: 1.00–1.067, *p* = 0.027) and the proportion of men (OR: 20.076, CI: 4.628–87.094, *p* < 0.001) were also risk factors associated with mortality in patients hospitalized for COVID-19 with elevated D-dimers.

**Figure 4:**
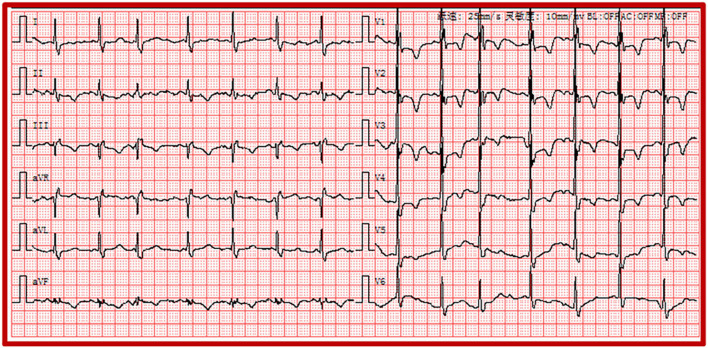
Electrocardiogram of the right ventricular strain including sinus rhythm, atrial premature, IRBBB, and T-wave inversions in the leads II, III and precordial leads V_1_-V_5_. IRBBB, incomplete right bundle branch block.

Recent investigations revealed that PE is closely associated with the pathogenesis of severe COVID-19 (20). The absence of anticoagulant therapy and elevation of D-dimer levels were both risk predictors for PE (21). The electrocardiographic Daniel score system has been widely applied to predict the severity of acute PE (22). Right ventricular strain manifested on ECG corresponded to the presence of right ventricular dysfunction with PE (22). The ECG of a 44-year-old man diagnosed with COVID-19 showed the right ventricular strain. The basic rhythm was sinus, accompanied by incomplete RBBB, inversion T waves in leads II and III, and precordial leads V_1_-V_5_ (Figure 4). The patient's ECG score was seven points according to the Daniel ECG scoring system. All ECGs of the patients with elevated D-dimer levels were scored according to the Daniel ECG scoring system (Table 4). The average number of deceased patients with COVID-19 was higher than that of recovered patients (2.57 ± 2.093 vs. 0.71 ± 1.28, *p* < 0.001).

**Table 4:** Comparison of ECG scoring between deceased and survived patients with elevated D-dimers.

**Elevated D-dimers**	**Cases**	**Daniel ECG scoring system**
Dead	47	2.57 ± 2.093
Survival	256	0.71 ± 1.28
*p*-value		<0.001

## Discussion

Our study is the first to demonstrate that ECG abnormalities combined with elevated D-dimer levels can estimate the risk of mortality in patients with COVID-19.

Our study observed that the rate of critically ill patients hospitalized with COVID-19 was 27.6% and the overall in-hospital mortality rate was 11.3%. Abnormal ECG manifestations were significantly more frequent in critically ill patients with COVID-19. The proportion of COVID-19 patients with elevated D-dimer levels was as high as 72.8%. D-dimer represents activation of the coagulation and fibrinolysis systems. Several studies showed that elevated D-dimer levels are closely associated with a poor prognosis of COVID-19 (21, 23, 24). Our studies similarly observed that elevated D-dimer levels contributed to higher mortality in patients with COVID-19 than in those without elevated D-dimer levels.

A systematic review of ECG findings in patients with COVID-19 revealed that the most prevalent ECG abnormalities were various patterns of ST-T abnormalities (25). Barmen et al. reported that ST-T abnormalities, including ST depression, T-wave inversion, and ST-T changes on admission ECG, were closely associated with the severity of COVID-19 (26). A study of 177 cases demonstrated that the proportion of ST-T changes on ECG was 49.15% in patients with PE (27). ST-T abnormalities are most common in precordial leads V_1_-V_3_ on ECG in PE (28). Figure 4 also shows that inversion T-waves occurred in the right precordial leads, which might be associated with the right myocardial strain. Similarly, in our study, ST-T abnormalities were the most prevalent ECG abnormalities in patients with COVID-19. ST-T abnormalities were more frequent in the critically ill group than in the severely ill group. Moreover, ST-T abnormalities were the most common ECG abnormalities in COVID-19 patients with elevated D-dimer levels. However, the relationship between elevated D-dimer levels and ST-T abnormalities in patients with COVID-19 has not been elucidated. We speculate that, in patients with COVID-19, ST-T abnormalities that widely occurred in those with elevated D-dimer were perhaps related to PE.

In those patients with COVID-19, the potential mechanisms of arrhythmogenesis were associated with multiple pathological conditions, such as hypoxia due to viral infection in lung tissues, impairment of host immune response, and myocardial ischemia (29). ST (heart rate >100 bpm) has been recognized as the most frequent supraventricular tachycardia in patients with COVID-19. The occurrence of ST (heart rate >100 bpm) was related to hypoxia, hypotension, hypoperfusion, and elevated body temperature in COVID-19 (7). In a study of 414 patients hospitalized for COVID-19, AF was the more frequent incident of arrhythmia (30). An Italian multicenter study reported that AF or AFL was found in nearly 22% of patients with COVID-19 (6). The occurrence of AF in COVID-19 might be associated with hypoxemia and could be permanent when accompanied by impaired pulmonary function (31). Furthermore, hypoxia and tachycardia have been proved to be the signs of PE (32). Atrial tachycardia has more frequently been associated with pulmonary or structural heart disease and hypomagnesemia (33). In our entire cohort, ST (heart rate >100 bpm) and atrial arrhythmias with rapid rhythms, including AF, AFL, and atrial tachycardia, were the two most common rhythm disturbances in patients with COVID-19. Moreover, the proportions of ST (heart rate >100 bpm) and atrial arrhythmias with rapid rhythms were higher in severely ill patients with COVID-19 than in critically ill patients.

The right myocardial strain has been shown to increase the incidence of circulatory shock and mortality in patients with PE (8). The main ECG signs of the right myocardial strain include ST (heart rate >100 bpm), S_1_Q_3_T_3_ pattern, IRBBB or CRBBB, and T-wave inversions (8). AF has also been found in >10% of patients with PE (34). However, the occurrence of AF was not associated with risk stratification in PE (34). D-dimer levels >1000 μg/L were associated with a high incidence of COVID-19 in patients with PE (16). In our study, a significant and direct association was observed between elevated D-dimer levels and ST (heart rate >100 bpm), AF, and atrial premature beats.

A multivariate analysis revealed that ECG signs, including ST (heart >100 bpm) and AF, were independent predictors of in-hospital death in patients with elevated D-dimer levels. Furthermore, on ECG, the existence of ST and AF might reveal a poor prognosis in patients with elevated D-dimer levels. In addition, patients' age and proportion of men were significantly associated with in-hospital death in patients with elevated D-dimer levels. According to our study, ECG abnormalities combined with elevated D-dimer levels were able to predict mortality in patients with COVID-19. Furthermore, elevated D-dimer levels were associated with diffused pulmonary intravascular thrombosis in COVID-19 but can occur due to many other conditions (35). Usually, the diagnosis of PE relies on imaging tests (such as computed tomography pulmonary angiography [CTPA]). However, the clinical use of CTPA is limited because of the adverse effects of the contrast medium. Due to the important diagnostic meanings of ECG and elevated D-dimer in PE, the occurrence of abnormal ECG and elevated D-dimer levels may be the potential risk factors for the increased mortality in COVID-19, which had a preference for PE or micro-embolism complications.

The Daniel scoring system has been extensively applied in predicting increased pulmonary arterial pressure by assigning points (0–21) to ECG components (22). We calculated the Daniel score in patients with and without elevated D-dimer levels. Similarly, the mean Daniel score in patients with elevated D-dimer levels was significantly higher than that in those without elevated D-dimer levels. Especially due to the outbreak of the COVID-19 pandemic, which limits critical medical resources, better use of limited CTPA resources seems to be required. Therefore, as an additional option, it seems that the combination of ECG abnormalities with elevated D-dimers is reasonable since it was able to predict the severity and mortality of COVID-19.

## Limitations

Our study has some limitations. First, we performed a retrospective study of patients with COVID-19 and specific laboratory investigations, such as CTPA, were not available. Moreover, CTPA examinations were not widely applied due to the limitation of critical medical resources during the outbreak of COVID-19 in 2020. Second, ECG signs dynamically changed in ECG monitoring during the process of disease duration, and some valuable and repeated manifestations were not possible.

## Conclusions

Elevated D-dimer levels are associated with an increased risk of critical illness and in-hospital mortality in patients with COVID-19. Moreover, the existence of ST (heart rate >100 bpm) and AF on the ECG may indicate a poor prognosis in patients with elevated D-dimer levels. ECG abnormalities (including ST [heart rate > 100 bpm] and AFL) in elevated D-dimer COVID-19 patients may indicate a higher risk of mortality.

## Data availability statement

The raw data supporting the conclusions of this article will be made available by the authors, without undue reservation.

## Ethics statement

The studies involving human participants were reviewed and approved by Institutional Review Board for human studies and the privacy of Tongji Hospital of Huazhong University of Science and Technology. Written informed consent for participation was not required for this study in accordance with the National Legislation and the Institutional Requirements.

## Author contributions

JC performed the statistical analysis and drafted the manuscript. YW collected the ECG recordings of patients with COVID-19. JW, LC, and QL collected clinical data from patients with COVID-19. XH and BW helped draft the manuscript. XL and HZ helped analyze data. XY and PZ conceived the study, participated in its design, and helped draft the manuscript. All authors contributed to the article and approved the submitted version.

## Funding

The supported funding was from the Interdisciplinary program of Wuhan National High Magnetic Field Center (Grant No. WHMFC22021122), Huazhong University of Science and Technology.

## Conflict of interest

The authors declare that the research was conducted in the absence of any commercial or financial relationships that could be construed as a potential conflict of interest.

## Publisher's note

All claims expressed in this article are solely those of the authors and do not necessarily represent those of their affiliated organizations, or those of the publisher, the editors and the reviewers. Any product that may be evaluated in this article, or claim that may be made by its manufacturer, is not guaranteed or endorsed by the publisher.

## Abbreviations

AF: atrial fibrillation
AFL: atrial flutter
CRBB: complete right bundle branch block
CTPA: computed tomography pulmonary angiography
CI: confidence interval
COVID-19: coronavirus disease 2019
ECG: electrocardiography
IRBBB: incomplete right bundle branch block
OR: odds ratio
PE: pulmonary embolism
ST: sinus tachycardia.
